# Emerging Role of Exosomes in Tuberculosis: From Immunity Regulations to Vaccine and Immunotherapy

**DOI:** 10.3389/fimmu.2021.628973

**Published:** 2021-04-01

**Authors:** Yin-Fu Sun, Jiang Pi, Jun-Fa Xu

**Affiliations:** Department of Clinical Immunology, Institute of Clinical Laboratory Medicine, Guangdong Provincial Key Laboratory of Medical Molecular Diagnostics, Guangdong Medical University, Dongguan, China

**Keywords:** *Mycobacterium tuberculosis*, exosomes, extracellular vesicles, innate immunity, immune evasion, vaccine

## Abstract

Exosomes are cell-derived nanovesicles carrying protein, lipid, and nucleic acid for secreting cells, and act as significant signal transport vectors for cell-cell communication and immune modulation. Immune-cell-derived exosomes have been found to contain molecules involved in immunological pathways, such as MHCII, cytokines, and pathogenic antigens. Tuberculosis (TB), caused by *Mycobacterium tuberculosis* (MTB), remains one of the most fatal infectious diseases. The pathogen for tuberculosis escapes the immune defense and continues to replicate despite rigorous and complicate host cell mechanisms. The infected-cell-derived exosomes under this circumstance are found to trigger different immune responses, such as inflammation, antigen presentation, and activate subsequent pathways, highlighting the critical role of exosomes in anti-MTB immune response. Additionally, as a novel kind of delivery system, exosomes show potential in developing new vaccination and treatment of tuberculosis. We here summarize recent research progress regarding exosomes in the immune environment during MTB infection, and further discuss the potential of exosomes as delivery system for novel anti-MTB vaccines and therapies.

## Introduction

By inducing over 1.2 million deaths and an additional 251,000 (Range: 223,000–281,000) human immunodeficiency virus (HIV)-positive deaths in 2019 (1), tuberculosis remains one of the most fatal public health threats in the world. Additionally, an increasing prevalence of drug-resistant and multidrug-resistant *Mycobacterium tuberculosis* (MTB) is seen under current anti-tuberculosis chemotherapy with limited efficacy, especially in underdeveloped and developing countries. Therefore, it is in urgent demand to develop novel vaccines and therapies against tuberculosis based on the in-depth understanding of the relationship between MTB and host immunity.

Innate immune cells, including macrophage and dendritic cells (DCs), are host cells for MTB, and perform most of the antibacterial activities during MTB infection. As the first barrier in MTB infection, alveolar macrophages (AMs) could produce inducible nitric oxide synthase (iNOS) that participates in the killing of MTB *via* the production of nitric oxide (2). Meanwhile, classical activation of macrophage induces polarization into the M1 antibacterial phenotype, with strong abilities in killing MTB by producing pro-inflammatory cytokines such as tumor necrosis factor-α (TNF-α) and interleukin-1 (IL-1) (3, 4). DCs, the “sentinels” of the immune system, are responsible for initiation of adaptive immune responses against MTB infection by migrating from infected lungs to local lymph nodes for T cell activation (5). Adaptive immunity is subsequently activated and engages in the host anti-mycobacterial activities, mediated by a range of different T cell and B cell subsets.

During MTB infection, a specific immune environment is formed when host immune cells interact with the pathogen at the infection site. The bactericidal immune responses of host cells are fine-tuned and balanced by multiple signal pathways to regulate immune cell functions. There are three widely accepted signal transduction methods among immune cells: direct immune cell contact (e.g. antigen presentation from DCs to T cells *via* direct MHC and T cell receptor contact); secretion of cytokines by immune cells to induce immune activities [e.g. interferon-γ promotes anti-MTB immunity (6); IL-10, transforming growth factor-β (TGF-β), and IL-35 can regulate immune function by manipulating inflammation (7, 8)]; and extracellular vesicle which include exosomes and microvesicles trafficking among immune cells. It has been demonstrated that exosomes from MTB-infected immune cells can regulate immune functions by transferring signal molecules into recipient cells (9, 10). In order to develop new anti-tuberculosis vaccine or therapy strategy based on exosomes, the underlying mechanisms of exosome in TB immunity need to be clarified. In this review, we discuss and summarize the immune regulator role of exosomes in the immune system in response to MTB which can extend our understanding of exosomes in TB immunity. Then we further discuss the possible role of exosomes in MTB immune evasion, as well as the protective role of exosomes to serve in anti-MTB vaccine.

## The Vector Role of Exosomes in Extracellular Signal Conduction

Exosomes, a type of nano-sized extracellular vesicle generated from multivesicular bodies (MVBs), contain constituents like protein, lipid, DNA, and RNA. They have been found to have unique physiological mechanisms and functions (11). Although exosomes are considered as waste carriers in autophagy process, more evidence has emerged to support cellular communication roles of exosomes (12). After summarizing several proteomic studies using different types of cells, Suresh Mathivanan has concluded that exosomes contain proteins like MVB biogenesis molecules [e.g. ALG-2 interacting protein X (Alix), and tumor susceptibility 101 (TSG101)], member RAS oncogene family (Rabs, facilitating exosome docking and fusion on the membrane), and annexins (assisting membrane trafficking and fusion events) (13). In recent years, researchers have also discovered various RNA contents in exosomes, including mRNA and miRNA, which can be transferred into recipient cells for cellular function regulations (14). Meanwhile, lncRNA is also found in exosomes that can regulate cellular functions. For example, HIF-1α-stabilizing lncRNA (HISLA) released from tumor-associated macrophages can enhance the aerobic glycolysis and apoptotic resistance of breast cancer cells (15).

After being released into extracellular environment, exosomes can be absorbed by different kinds of cells, in which they will perform cellular signal transduction and communication in two main ways. The first one is the binding of exosomes to specific cell membrane molecules. For example, genetically engineered chimeric antigen receptor T lymphocyte cells (CAR-T cells) can secret exosomes with chimeric antigen receptor (CAR) protein on the surface, which can inhibit tumor growth through binding to specific tumor antigens (16). The second way is that exosomes can be transferred into the target cells through endocytosis. Upon entering, exosomes can release their cargo into the target cells and execute biological functions (17). Therefore, exosome performs important vector role in facilitating cell-cell signaling communication by using its contents to regulate various cellular functions.

## Double Roles of Exosomes in Anti-infection Immunity

When infection occurs, the innate immune system tries to kill the pathogen as well as presents antigens to prime the adaptive immune system for more effective pathogen clearance. Exosomes have been found to contain plenty of immune-regulating molecules with functions such as indirect activation of T cells by DC-derived exosomes to help recipient cells to confer HIV resistance (18, 19). It has also been discovered that DC can release exosomes with MHC-I/peptide complexes for other naïve DCs to uptake, eventually helps to prime CD8+ T lymphocyte cells (20). In tumor environment, DC-derived exosomes containing TNF, Fas ligand (FasL), and TNF-related apoptosis-inducing ligand (TRaIL) could lead to tumor cell apoptosis (21) and activate natural killer cells *via* TNF superfamily ligands for enhanced tumor inhibition (22). Macrophages secrete cytokines to create a pro-inflammatory environment against the pathogen, during which the exosomes play critical roles. It has been found that multiple intracellular pathogens, such as MTB, *Bacillus Calmette-Guerin* (BCG), *Salmonella typhimurium*, and *Toxoplasma gondii*, can induce infected macrophages to secrete exosomes with pathogen-associated molecular patterns (PAMPs). Those exosomes are in turn transferred into uninfected macrophages to be activated through Toll-like receptor (TLR) and myeloid differentiation factor 88-dependent (MyD88) pathway (23, 24). More interestingly, exosomes from T cells could be transferred into DC and induce more resistant DC antiviral responses *via* the cyclic GMP-AMP synthase/stimulator of interferon response cGAMP interactor 1 (cGAS/STING) cytosolic DNA-sensing pathway and *via* the expression of interferon regulatory factor 3 (IRF3)-dependent interferon regulated genes (25).

Immune evasion pathways are found both in MTB infection and other pathogen-induced diseases, where exosomes are closely engaged in immune attack regulation, creating a pro-bacteria or pro-virus environment. In Hepatitis C virus (HCV) infection, hepatocyte-derived exosomes containing TGF-β could promote the expansion of T follicular regulatory (Tfr) cells in healthy subjects’ PBMCs, inhibiting the function of T cells and B cells, leading to an environment in favor of HCV survival (26). This means that exosomes can also act negatively during immune regulation in infectious disease and its vector role in the immune system is a double-edged sword. Another study found that Newcastle disease virus (NDV)-infected HeLa cell-derived exosomes can promote NDV replication by three miRNA inside, which were associated with enhancing NDV-induced cytopathic effects and suppressing IFN-β gene expression (27). Therefore, exosomes could have either beneficial or harmful properties during infection, depending on the type of their regulatory molecules.

## Exosomes Function as Protective Stimulators During MTB Infection

MTB infection stimulates immune cells to secrete different kinds of exosomes which act as pre-stimulators for immune system even before the activation of DCs, macrophages, T cells, and B cells. Meanwhile, exosomes can activate inflammatory and autophagy signaling pathways in host cells, which can not only enhance the anti-MTB immunity by helping to kill intracellular MTB, but also prepare uninfected immune cells for upcoming MTB infection.

## MTB-Infected Macrophages-Derived Exosomes Stimulate Naïve Macrophages and Induce Secretion of Pro-Inflammatory Cytokines

Macrophages, the first in defense line in face of MTB infection, could trigger intracellular downstream inflammatory signaling pathways for anti-MTB activities, such as the activation of mitogen-activated protein kinase (MAPK) and nuclear factor kappa B (NF-κB) signaling pathways (28). Meanwhile, they can activate the downstream transcription factors to start translation of target genes and initiate inflammatory responses. Pattern recognition receptors (PRRs) and PAMPs are critical in inflammation, especially TLRs, which can induce releasing of pro-inflammatory cytokines, iNOS, and antimicrobial peptides *via* MyD88 (29, 30). Interestingly, it is found that exosomes secreted by MTB-infected macrophages also contain bacterial-derived RNA, providing strong evidence for exosome-induced anti-MTB immune responses. Notably, MTB peptides are also found in serum extracellular vesicles from persons with latent tuberculosis infections (31, 32). These studies strongly suggest that exosomes from MTB-infected cells could act as PAMPs, which induce naïve macrophage activation and would be beneficial for the anti-MTB immunity.

By regulating pro-inflammatory responses, exosomes from infected macrophages is an essential force against MTB infection as they can stimulate a higher level of cytokines and chemokines production from bone marrow-derived macrophages (BMMs) than from resting macrophages (9). Furthermore, Singh et al. tested the ability of exosomes from MTB-infected macrophages to influence the immune cells using Transwell system, and found that exosomes-treated macrophages could induce stronger transmigration ability than the resting cells. The researchers also extracted exosomes from BCG-infected mice serum, and found them to activate macrophages as pro-inflammatory phenotype, and recruit macrophages and CD11b^+^ cells to the lungs of mice (9). This study provides direct evidence for the hypothesis that exosomes from infected macrophages could activate uninfected macrophages and recruit other immune cells. Additionally, another research revealed that extracellular vesicles (EVs) secreted from MTB-infected macrophages or mice could activate endothelial cells, which indicated that exosomes might play comprehensive roles during immune activation (33).

A subsequent study demonstrated that exosomal RNA from MTB-infected cells could stimulate a higher level of cytokine and chemokine, and induce more significant apoptosis in macrophages than that by exosomal RNA from uninfected cells (31). Moreover, exosomes from BCG-infected macrophages could activate TLR/MyD88-dependent proinflammatory pathways in BMMs, associated with the lipoarabinomannan (LAM) and the 19-kDa lipoprotein contained in the exosomes that are capable of promoting TNF production (23). With the above evidences, it is suggested that MTB-infected macrophages-derived exosomes could serve as naïve macrophages activating components by regulating pro-inflammatory responses.

## Exosomes Present Antigen to Activate Adaptive Immune System During MTB Infection

CD4^+^ T lymphocyte cells play an important part in activating macrophages against MTB infection and inducing apoptosis in infected cells *via* IFN-γ, which is mainly secreted by T helper type-1 (Th1) cells. To initiate adaptive immunity, antigen-presenting cells (APCs) load exogenous antigens on MHC-II molecules *via* endocytic pathway and increase the surface expression of T cell costimulatory molecules (34). Exosomes from MTB-infected macrophages have been found to contain various mycobacterial proteins such as Antigen 85-C and early-secreted antigenic target of 6-kDa (ESAT-6), all important antigens for T cell activation against MTB infection (35). Exosomes secreted from MTB culture filtrate protein (CFP)-treated macrophages could activate macrophages, DCs, as well as naïve T cells *in vivo* to mediate protective responses with high expression of antigen-specific IFN-γ and IL-2, resulting in lower MTB loads in the lungs of mice (36). Such *in vitro* and *in vivo* studies demonstrated the role of exosomes in the process of MTB antigen presentation to T cells to activate T cell protective responses against MTB.

DC derived-exosomes have been found to contain MHC and costimulatory molecules, indicating potential ability of exosomes in antigen presentation. Moreover, antigen-bearing exosomes from DCs can activate antigen-specific naïve CD4^+^ T lymphocyte cells *in vivo*, but cannot induce the CD4^+^ T lymphocyte cells activation without mature DCs *in vitro*, suggesting DC’s ability to present antigens without contacting bacteria by manipulating exosomal antigen-MHC complex (19). The ability of antigen presentation and naïve T cell activation can also be found in exosomes secreted from MTB-infected macrophages, which can induce the activation of DCs and the generation of memory CD4^+^ T lymphocyte cells and CD8^+^ T lymphocyte cells (10).

Additionally, some factors are reported to influence the antigen-presenting role of exosomes. Ramachandra et al. reported that MTB synergized with ATP to induce a more potent release of exosomes containing MHC-II molecules, which were capable of antigen presentation (37). In Rab27a-knockout mice, exosome concentration decreased as Rab27a mediated MVB docking to the plasma membrane, which caused further diminution of T cell responses, leading to increased bacterial loads in the lungs of mice. The reduction of exosomes also led to decreased trafficking of antigens (Ag85A) to exosomes, explaining the reduced priming ability of exosomes (38). It has been clearly demonstrated that MTB-infected immune cell-derived exosomes play important roles in both innate and adaptive immunity, and any disruption of exosomal functions would impair part of the anti-tuberculosis immune protection.

## Exosome Might Stimulate Autophagy for MTB Clearance

Autophagy, a form of cellular metabolism involved in innate immunity, is a pathway for the clearance of cellular waste substances or organelles. Low energy, malnutrition, and stress could all contribute to the activation of intracellular autophagy signaling pathways, which degrades the phosphorylation of mammalian target of rapamycin complex 1 (mTOR1), followed by the initiation and continual expansion of membrane to form autophagosomes (39). By further fusion with the lysosomes, autophagosomes would turn into autolysosomes, which have strong abilities in eliminating damaged organelles, protein aggregates, and intracellular pathogens. For host cells, autophagy has been recognized as an important innate defense mechanism against intracellular MTB, and in this way, host cells are sacrificed for effective attack on MTB (40). The autophagy-defective mice are found to show excessive inflammation in lungs and increased bacterial burden, indicating the critical roles of autophagy against MTB infection (41).

A growing number of studies have demonstrated that the antibacterial function of EVs is mainly achieved through modulating autophagy. EVs extracted from infected and uninfected macrophages can both significantly reduce the bacterial loads in mice lungs. However, EVs from uninfected macrophages reduce the bacterial load by production of C-C motif chemokine ligand 2 (CXCR2), while EVs from MTB-infected macrophages produce TNF-α (42). These results indicate that the contents of EV could inhibit MTB both *in vitro* and *in vivo*. EVs secreted from MTB-infected human neutrophils can induce the production of pro-inflammatory cytokines such as TNF-α, IL-6, and superoxide anion to inhibit the intracellular MTB in human macrophages (43). Meanwhile, autophagy is significantly upregulated, and higher expression of autophagy-related marker LC3-II is seen in infected macrophages co-localizing with MTB (43). These results further confirm that EVs from infected innate immune cells can upregulate autophagy for intracellular MTB clearance to lower the intracellular bacterial load. MTB RNA is found to be contained in EVs from infected BMMs, which could pass through host nucleic acid-sensing pathways (RIG-1/MAVS/TBK1/IRF3) to further activate autophagy in MTB-infected macrophages with the co-localization of LC3-II and MTB for MTB elimination (44).

**Figure 1:**
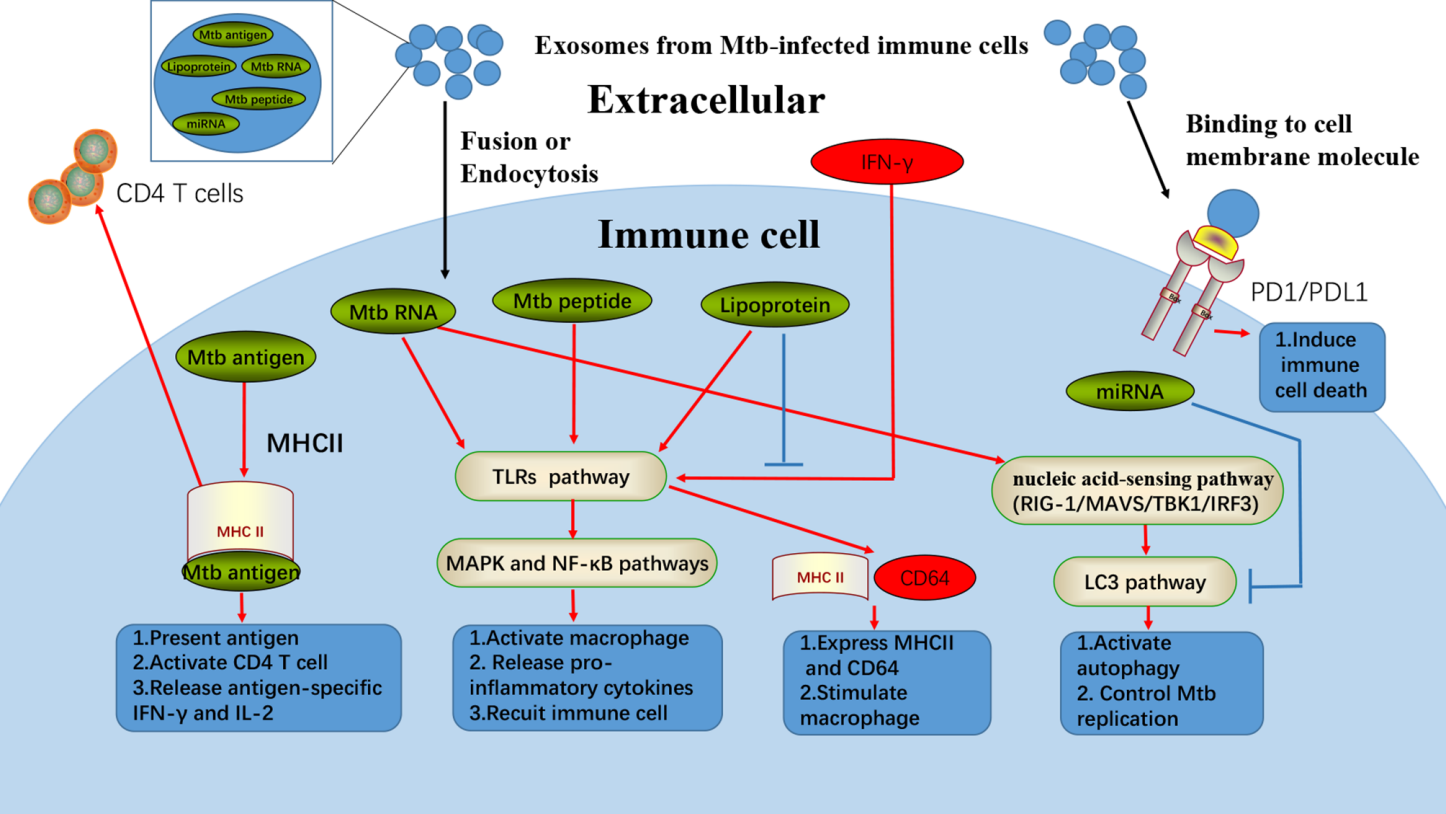
Exosomes from Mtb-infected immune cells could induce multiple cellular responses. Exosomes from Mtb-infected immune cells contain various regulatory materials, such as Mtb antigens, Mtb RNA, Mtb peptide, lipoprotein, and miRNA. In fusion with cell membrane or passing through endocytosis, exosomes can release some Mtb specific contents to induce different anti-Mtb responses in immune cells. Mtb antigens from exosomes can be transferred into uninfected-DC, where the antigens can combine with MHCII for antigen presentation and activating the adaptive immune systems. Mtb RNA, peptide, and lipoprotein from exosomes are found to be responsible for exosomes induced-inflammation, which are in strong association with TLRs, MAPK, and NF-κB pathways. Furthermore, Mtb RNA from exosomes can also stimulate macrophage autophagy through nucleic acid-sensing pathways (RIG-1/MAVS/TBK1/IRF3). However, exosomes from Mtb-infected immune cells can also inhibit anti-Mtb functions. For example, lipoprotein from exosomes can suppress the IFN-γ induced MHCII and CD64 expression. Besides, miRNA from exosomes can inhibit autophagy of immune cells, while Mtb infection as well as PD1/PDL1 from exosomes can also act as immune inhibitor.

As a member of EV family, exosomes have also been found to be in strong association with autophagy. The newly discovered evidence indicates that exosome biogenesis is closely related to autophagy linked by the endolysosomal pathway (45). Exosomes secreted from miR-181-5p-modified adipose-derived mesenchymal stem cells are found to prevent liver fibrosis *via* autophagy activation, showing its capability to activate autophagy (46). These results reveal the potential bidirectional regulation effects between exosomes and autophagy. This regulation is mediated by the unique biogenesis mechanism involved in autophagy, as well as by the MTB components and membrane molecules contained in exosome that might induce autophagy for MTB clearance (47, 48). As shown in **Figure 1**, we summarize that exosomal contents from infected innate immune cells, including RNA, antigens of MTB, and some MTB peptides, would trigger multiple antibacterial immunological functions *via* a series of traditional pathways. Additionally, we consider that exosomes that inherit the functions of their parent cells could perform cargo delivery function to induce immune functions, such as inducing autophagy. However, almost all published works focused on the effects of exosomes on other innate immune responses. Further studies are needed to explore how exosomes act on autophagy of host cells against MTB infection.

## Exosomes Contribute to Immune Evasion of MTB

Exosomes secreted from infected immune cells can induce stronger anti-MTB activities in different immune cells, supporting the potential use of exosomes for the development of vaccine or immunotherapy strategies. However, as one of the cleverest bacteria, MTB is continuously altering the environment for immune evasion. Early infection defense can be induced by constituents from MTB that interact with host protein and interfere with host immune cells. However, in long-term infection, these constituents from MTB could be harmful. Moreover, host proteins and miRNA are changed during MTB infection and can facilitate immune evasion of MTB. These molecules can be transferred to different immune cells by exosomes, creating an environment more prone to MTB immune evasion.

Exosome can induce various biological or immunological effects depending on the cargo inside. Pramod et al. identified 41 mycobacterial proteins present in exosomes released from MTB-infected J774 cells, including some very important MTB antigens. Many of these identified proteins were characterized as highly immunogenic, especially Ag85b, which was widely used for TB vaccine development (35). Small EV proteome isolated from active tuberculosis (ATB) were found to carry host proteins in TB-positive patients, which showed significant deregulation and could be useful in developing alternate host-directed therapeutic interventions (49). Using multiplexed multiple reaction monitoring mass spectrometry (MRM-MS), Nicole A et al. analyzed exosomes isolated from human serum samples obtained from culture-confirmed active TB patients and found 76 peptides representing 33 unique MTB proteins (50). Twenty of the 33 proteins detected were found in the exosomes of TB patients, including several peptides from eight important MTB proteins, which were known to contribute to the intracellular survival of MTB (50). These MTB and host proteins, as well as the molecules that we have summarized above in exosomes, allow exosomes to directly interact with different immune cells with immune regulation effects. Here we summarized the exosome sources, different isolation methods, and culprit cargos from the quoted studies (**Table 1**).

**Table 1:** Summary of the exosome sources, different isolation methods, and different culprit cargo from the studies.

Exosome sources	Isolation methods	Identified cargo	Author
MTB-infected macrophages	Sucrose-gradient ultracentrifugation, Ultracentrifugation	MTB components Lipoarabinomannan and the 19-kDa lipoprotein	Bhatnagar et al. (23)
MTB-infected macrophages	Ultracentrifugation	MicroRNA, MRNA	Singh et al. (31)
Serum from persons with latent tuberculosis infection	Exoquick	MTB peptides (Ag85c, DnaK, HspX, Ag85A et al.)	Mehaffy et al. (32)
MTB-infected and CFP-treated macrophages	Sucrose-gradient ultracentrifugation	Mycobacterial proteins (Antigen 85-C, GInA, 19 kDa Lpqh et al.)	Giri et al. (35)
MTB CFP-treated macrophages	Exoquick, Ultracentrifugation	19 kDa lipoprotein	Cheng et al. (36)
MTB-infected macrophages	Exoquick, Ultracentrifugation	MTB RNA	Cheng et al. (44)
Serum of tuberculosis patients	Exoquick, Sucrose-gradient ultracentrifugation	Host proteins (KYAT3, SERPINA1, HP, and APOC3)	Arya et al. (49)
Serum of tuberculosis patients	Exoquick	MTB peptides (Antigen 85B, Antigen 85C, Apa, BfrB, GlcB, HspX, KatG et al.)	Kruh-Garcia et al. (50)
Urine of tuberculosis patients	Centrifugation	Lipoarabinomannan and CFP-10	Dahiya et al. (51)
MTB-infected macrophages	Sucrose-gradient ultracentrifugation	Lipoproteins	Singh et al. (52)
Serum of tuberculosis patients	Ultracentrifugation	MicroRNA (hsa-miR-1246, hsa-miR-2110, hsa-miR-370-3P, hsa-miR-28-3p, and hsa-miR-193b-5p et al.)	Lyu et al. (53)

Although specific proteins and peptides from MTB have been identified, exosome-induced immune regulation effects are still under investigation because MTB components can induce complicated effects. For example, LAM from MTB has been found in the urinary extracellular vesicles of tuberculosis patients (51) and also in exosomes isolated from the broncho alveolar lavage fluid (BALF) of BCG-infected mice (23). LAM from MTB or other pathogenic mycobacteria is a high-molecular-mass, amphipathic lipoglycan with a defined critical role in mycobacterial survival during infection (54). It is thought to show both active and passive protection against TB (55). Although LAM shows ability to activate immune cells, Nicole P et al. also demonstrated that LAM from MTB could reduce the expression of chemokine receptors CXCR2 by a mechanism that involved the activation of p38 MAPK (56). As CXCR1 and CXCR2 determines the functional properties of granulocytes, the LAM inside exosomes from MTB-infected immune cells suggest the ability of the host to limit inflammation induced by granulocytes after MTB infection. Despite the anti-MTB effect brought by exosomes, MTB has been menacing human health throughout history. With a wide variety of immune evasion mechanisms and its strong pathogen-host interaction ability, MTB can also have exosomes act negatively (57). Several studies have proved that MTB could influence macrophage functions such as apoptosis, autophagy, and MTB-lysosome fusion by some of the MTB components that interfere with anti-MTB effects (58, 59). An affinity tag purification mass spectrometry (AP-MS) study has demonstrated the interaction map between MTB components and host cell proteins, and found that a lot of the components can interact with host proteins by regulating various cell functions (60). More importantly, exosomes from MTB-infected macrophages are found to induce a decline of IFN-γ-induced MHC-II and CD64 expression through TLR2 and Myd88 pathway, partially *via* the lipoproteins in exosomes (52). This study reminds us that exosomes can act negatively in defending MTB infection, which means that the immune regulation roles of exosomes can be either inhibiting MTB infection or promoting MTB infection. Based on these results, we hypothesize that at the early stage, the cargos coming from MTB inside the exosomes could activate the anti-MTB effect. However, MTB can survive in macrophages, and after the early stage, an increasing amount of MTB components are able to interact with host proteins and interfere with the activated immune function. We further speculate that the exosome-induced immune suppression of IFN-γ-induced MHC-II and CD64 expression (52) is like MTB components-induced immune evasion, which mainly happens at the post-infection stage. Therefore, exosomes can suppress the activated macrophages’ function instead of acting as an immune stimulator. For our understanding, the cargos inside the exosomes would be a very good anti-TB immune stimulator in inactivated immune cells. However, as exosomes are part of the existing signal conduction pathways of cells during MTB infection, they show two-sided effects on TB immunity as the pathogen might pretend to use these exosomes for their immune escape. Therefore, these exosomes from MTB-infected cells might be anti-MTB vaccine candidates due to their ability to activate anti-MTB immunity in rest of the immune cells. However, they might also block cellular antibacterial immunity by “hijacking” immune cells.

Furthermore, RNA in exosomes, especially miRNA for regulating host cell functions, plays important roles in MTB evasion. RNA sequencing provides a powerful strategy to explore the miRNA in exosomes from MTB-infected macrophages and plasma from tuberculosis patients, which are predicted to be closely linked with the metabolism and energy production-related pathways (53, 61). MiR-18a, a type of miRNA also found in exosomes, is upregulated to promote mycobacterial survival in MTB-infected macrophages by inhibiting the autophagy pathways (62). In MTB infection, individual miRNA alternation might induce cellular function changing as miRNA could regulate gene expression post-transcriptionally. Furthermore, miRNA can influence multiple antibacterial functions of different immune cells (such as macrophages, DCs, and CD4^+^ T lymphocyte cells) by regulating apoptosis, autophagy, and polarization (63, 64), making miRNA an important host material for immune regulation and a pathway for MTB immune evasion. As miRNA contained in exosomes can be transferred to different cells of the immune system, harmful immune functions can also be shared by MTB-benefiting miRNA.

Moreover, as a star molecule in immune suppression, programmed cell death 1 ligand 1 (PD-L1) is also found in exosomes, which suppresses the function of CD8^+^ T lymphocyte cells and facilitates tumor growth (65). Increased programmed cell death protein 1 (PD-1)/PD-L1 expression, which increases the macrophage susceptibility to MTB-specific CD8^+^ T lymphocyte cells, can lead to cell deaths (66), and this is already proved in MTB infection. This indicates that the exosomal PD-L1 might also contribute to the immune evasion of MTB. Taking all the results into account, exosomes from MTB-infected cells could also act as an accomplice for MTB immune evasion by delivering components benefiting MTB survival, as shown in **Figure 1**. More attention should be paid to the exploration of MTB-benefiting components in the exosomes, which would help develop novel immunotherapy strategies to restrain MTB infection.

## Exosomes for Vaccine and Immunotherapy Development During MTB Infection

The full role of exosomes during MTB infection is still to be revealed. We should be exploring how exosomes help MTB escape from the immune attack and also trying to utilize the antibacterial function of exosomes for TB treatments. Exosomes carrying mycobacterial antigens can significantly protect mice against MTB infection, indicating the potential of exosomes in serving as a novel cell-free vaccine targeting MTB infection (36). EVs from MTB-infected BMM can induce autophagy for *in vitro* MTB killing and also decrease mycobacterial burden in the lungs of mice with lower tissue damage (44). These studies strongly suggest that exosomes may be a candidate vector for vaccine or drug delivery.

In theory, exosomes are promising delivery tools for vaccines and treatments thanks to their natural nano-size lipid membrane structures. The vaccine based on exosomes with specific antigens inside can activate multiple immune responses *via* antigen presentation pathways. For example, exosomes loaded with tumor-associated-antigens can activate adaptive immunity as well as improve antitumor efficacy both *in vivo* and *in vitro* (67, 68). In another study, researchers developed a novel vaccine based on Ag85B-ESAT-6 fusion protein expressed in exosomes, which could be further introduced for activating antigen-specific INFɣ-secreting T lymphocytes in the lungs and spleen (69). Moreover, the anti-MTB immunity induced by exosomes-based vaccine can also be improved by costimulatory molecules in exosomes. Hao et al. established ovalbumin (OVA)-pulsed exosomes from dendritic cells to target CD4+ T cells as a cancer vaccine, which also successfully stimulated CD8+ cytotoxic T lymphocyte (CTL) responses for enhanced antitumor immunity (70). They found that the exosomal CD80 (70) and exosomal CD40L (71) were crucial in the development of functional memory CTLs. Interestingly, it was also found that exosomes observed in BALF were expressing MHC class I and II, CD54, CD63, and the costimulatory molecule CD86, suggesting that exosomes might have a role in antigen delivery or immune regulation during airway antigen exposure (72). Using MTB-infected antigen-presenting cell-derived exosomes as vaccine not only can realize antigen presentation but also express costimulatory molecules which can stimulate strong anti-MTB immunity.

Additionally, exosomes can act as adjuvants that stimulate immune responses, which can improve vaccine efficiency by mediating the immune environment of adaptive immune system. It has been proven that hepatitis B recombinant antigen (HBsAg), combined with exosomes from LPS-stimulated macrophages, can induce pro-inflammatory cytokine expression and antibody release similar to HBsAg alone (73). However, exosomes could have further immunomodulatory effects on the cellular immune response, highlighted by the enhancement of IFN-γ secretion as Th1 cell responses (73). Another study also demonstrated that exosomes from TGF-β1-silenced leukemia could promote DC maturation and their immune function, backing up the roles of exosomes as adjuvants to establishing enhanced anti-tumor immunity (74). Exosomes have the abilities, inherited from parent cells, of boosting anti-MTB immunity through traditional pathways, including macrophages, DCs, and neutrophil granulocytes. These anti-MTB immune responses can be triggered by some regulatory proteins in exosomes, such as heat-shock protein (75) and PAMP (23). These findings demonstrated the possibility of using exosomes from infected innate immune cells for specific antigen loading, costimulatory molecules stimulating, and immunity boosting, which would be beneficial for vaccine development.

**Figure 2:**
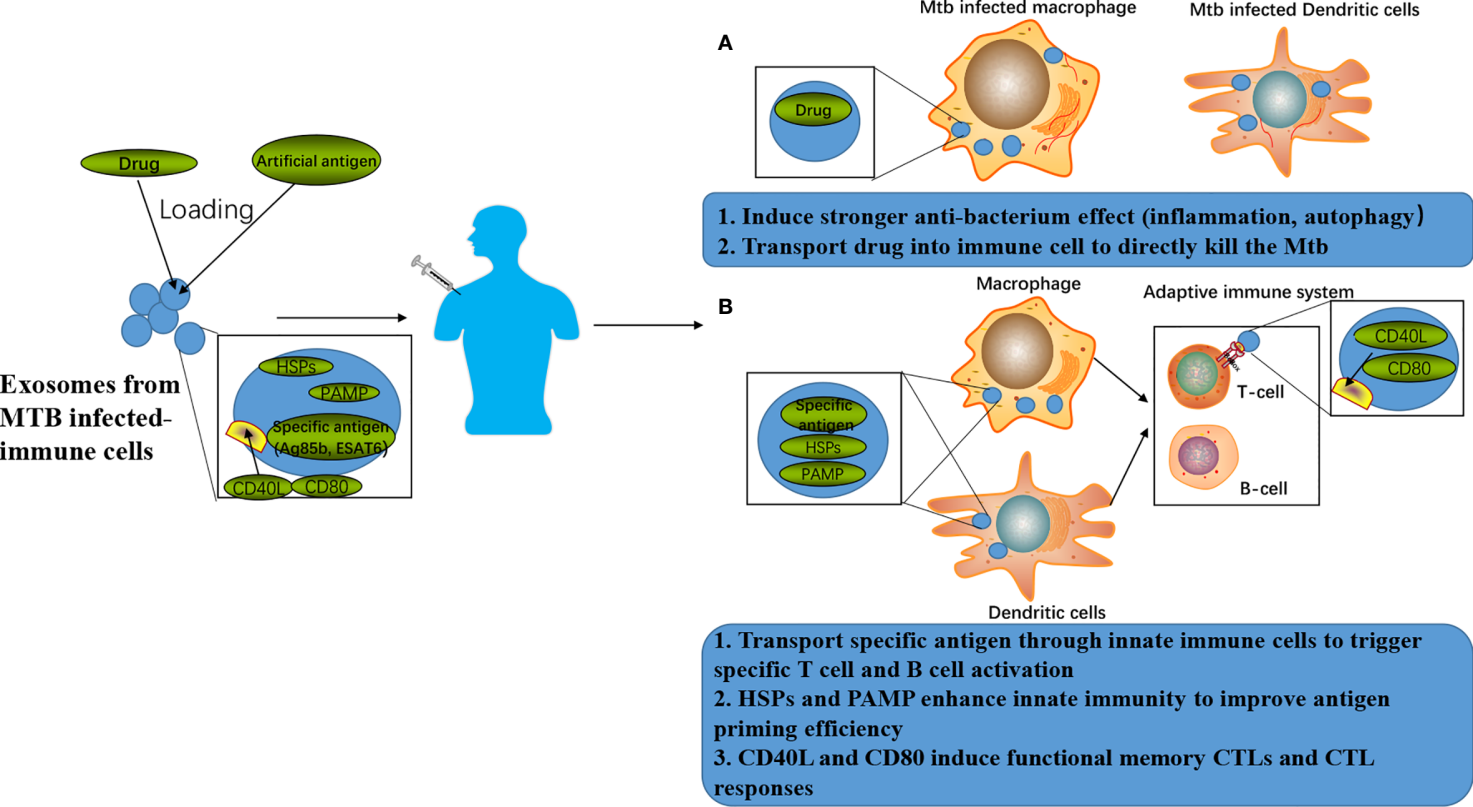
Exosomes-associated anti-TB strategies. **(A)** Exosomes can be loaded with drugs to achieve enhanced anti-TB therapy by combining the improved killing efficiency induced by the drugs and anti-TB responses induced by the exosomes. **(B)** Exosomes can be loaded with specific antigens to achieve enhanced anti-TB immune responses by combing the T cell and B cell activation induced by the specific antigen as well as anti-TB responses induced by the exosomes.

Apart from acting as components in immunological regulation, exosomes can also be applied to direct drug delivery, which help overcome the major challenges of drug treatment, i.e., delivery of cargos across impermeable biological barriers and improvement of the target effects and pharmacokinetics of drugs (76). As a novel drug delivery system, exosomes isolated from M1 macrophages have been proved to enhance the cytotoxicity of Paclitaxel (PTX) in cancer cells and show stronger antitumor efficacy (77). A majority of published studies focused on using exosomes for anticancer drug delivery, inspiring the application of exosomes in anti-MTB drug delivery, which could potentially improve the current TB chemotherapy efficacy. Furthermore, certain components with antibacterial regulation effects, such as non-coding RNA, can also be loaded into exosomes for anti-MTB treatment. Endowed triply for its potentials in novel vaccine, immune therapy, and chemotherapy strategy developments as shown in **Figure 2**, exosomes bring hope for anti-MTB treatments.

Lipids are critical components of exosomal membranes, and it is well-known that some specific lipids are even more enriched in exosomes compared to their parent cells. Therefore, the use of exosomes for delivery can be considered as using “natural lipid nanoparticles.” As a “competitor” against the current lipid nanoparticles (nanolipids), it would be crucial to compare the properties of exosomes with normal lipid nanoparticles, which would benefit our understanding of the advantage and limitation of exosomes as delivery systems. As shown in **Table 2**, firstly, exosomes have natural immunogenicity and antigenicity depending on the cargo loaded inside, while normal lipid nanoparticles do not have similar abilities. This property can distinguish between lipid nanoparticles and exosomes, and the immune regulatory function of exosomes can be mediated by controlling the parent cells. Based on these properties, exosomes can be used as natural adjuvants, natural vaccines, or natural immunomodulators. Secondly, the cell membrane structure of exosomes can protect the loaded drug, protein, or RNA from the extracellular environment with less rejection than chemosynthetic lipid nanoparticles, providing much safer transportation environment (78). Last but not least, exosomes have the ability to pass through the blood-brain barrier without further modification (79), while lipid nanoparticles can require specific chemical modification (80).

**Table 2:** Advantages and limitations of exosomes compared with nanolipids (lipid nanoparticles) for vaccination.

	Advantages	Limitations
Nanolipid-based vaccination	1. Easy to be modified 2. Controllable size and shape 3. Long-term stability 4. Inexpensive expense 5. Easy for quality control	1. No natural antigenicity or immunogenicity 2. Need specific modification to pass blood-brain barrier
Exosome-based vaccination	1. Natural antigenicity and immunogenicity 2. Natural immunomodulators 3. Better protection of inside cargo by cell membrane structures 4. Natural ability to pass blood-brain barrier	1. Difficult in quality control 2. Unpredictable content insides 3. Difficult to store and transportation 4. MTB constitutes docking pathway unclear 5. Modification techniques immaturity 6. Expensive expense

However, we cannot deny that limitation exists in the application of exosomes as delivery vectors. The quality control of exosomes is still a big challenge to be addressed. The contents inside exosomes always change along with the functions and status of parent cells, leading to an unpredictability for both cargo delivery and subsequent effects. Besides, as the structure of exosomes is similar to the cell membrane, some environmental factors such as pH and temperature, as well as preparation procedures such as ultracentrifugation and freezing-thawing, might introduce unexpected damage to exosome structures, subsequently affecting the quality of exosomes (81). However, chemosynthetic lipid nanoparticles can be prepared with constant quality, which is also easy to be further modified and stored. Moreover, the types of nanoparticles-induced responses in recipient cells are constant, while exosome-induced cellular responses are much more complicated, unpredictable, and uncontrollable. For example, exosomes from MTB-infected macrophages can induce multiple anti-MTB effects, but at the same time promote the immune escape of MTB. Furthermore, the detailed mechanism that parent cells sort out MTB components and dock them into exosomes remains unknown, which also introduce unpredictability into their delivery actions. Additionally, the modification techniques of exosomes are still very limited, rendering the surface modification of exosomes difficult. Finally, it is also worth noting that the high cost of exosome preparation would also be considered a critical limitation compared with lipid nanoparticles.

**Figure 3:**
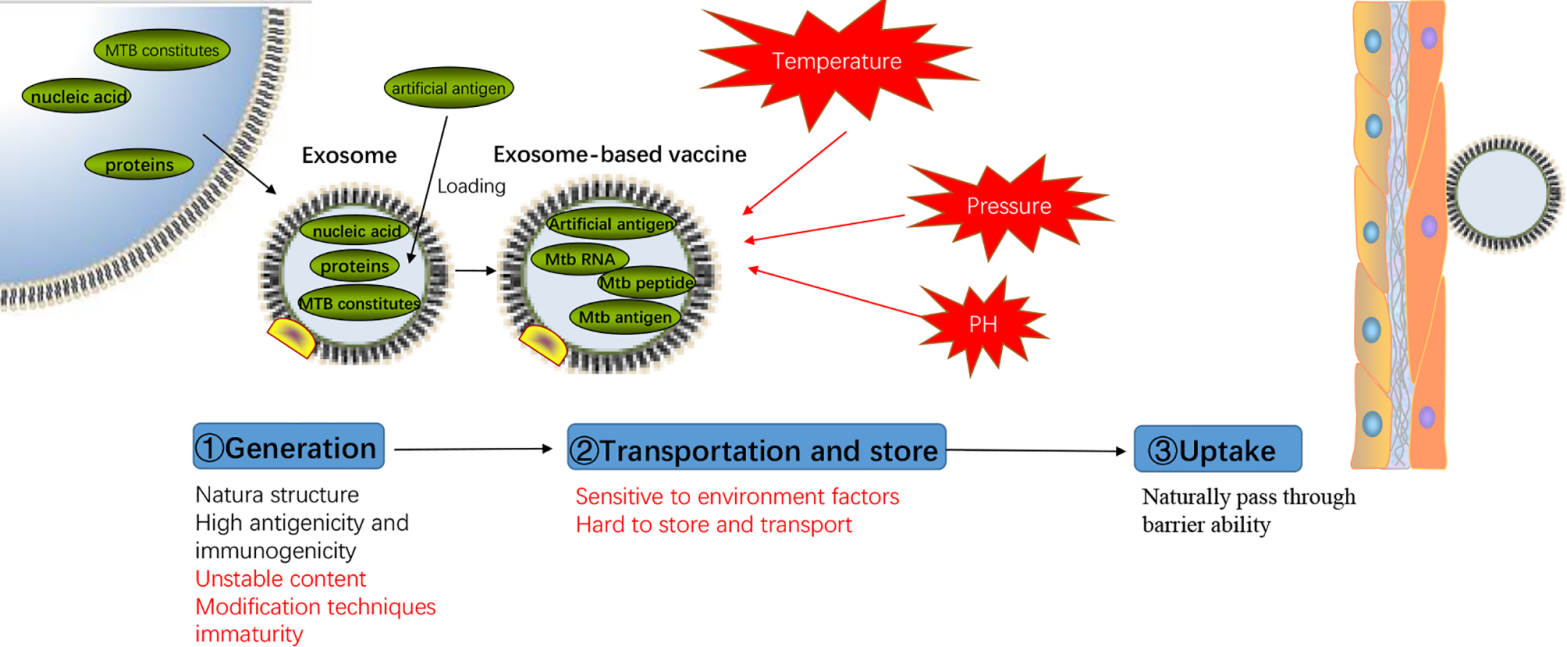
Advantages and disadvantages of exosomes-based vaccine. Representation of the process of exosomes-based vaccine generation, transportation, storage, and uptake with the advantages and disadvantages (mark in red) of each step.

Taking all these considerations into account (as shown in **Figure 3**), although exosomes show multiple limitations for vaccination, their advantages, including their natural antigenicity and immunogenicity to regulate immune responses, provides an attractive blueprint for more powerful vaccine developments. Thus, to extend the application of exosomes as effective drug delivery systems for vaccination and therapy, it would be critical to clarify the underlying mechanisms involved in the formation and cargo loading of exosomes in parent cells. Additionally, more attention should be paid to the quality control, preparation, and modification methods of exosomes to obtain homogeneous, constant, storable, and more functional exosomal products, benefiting the use of exosomes for novel vaccination and therapy strategy development.

## Conclusion and Remarks

In MTB infection, exosomes from the infected immune cells have double inherent immune regulation functions their parent cells with double-edged sword regulation effects on anti-MTB immunity. However, previous studies only focused on exosomes from innate immunity cells, and little is understood about the exosomes secreted from adaptive immune cells, which are of more importance in facing MTB infection. Therefore, exploring potential effects of exosomes from innate immune cells on MTB infection is helpful in developing new vaccination and therapy. Up to now, diagnosis, vaccination, and treatment of TB, especially drug-resistant TB, remains major clinical challenges. Different RNA molecules have been found in exosomes after MTB infection, which shed new light on the potential role of exosomal RNAs as novel TB biomarkers for developing the next generation of TB diagnostic strategy and relevant studies have already begun (82–84). Exosomes have shown strong potential in delivering vaccine components (proteins, peptides, and RNA) in different infectious disease, showing the potential to provide a more effective vaccine strategy for TB. Albeit the limited knowledge regarding the drug delivery roles of exosomes for anti-MTB treatment, the strong ability of macrophages to internalize the nanosized system allows macrophage-targeted drug delivery for anti-MTB treatment. However, careful consideration is still in need when applying exosomes as drug delivery systems as they also have negative roles in immune function. Further surface functionalization of exosomes with specific ligands would increase the targeting effects against specific cell types (85), which reminds us that some ligands with macrophage targeting effects could benefit the anti-MTB therapy by exosomes functionalization and drug delivery. Most importantly, designing cell systems to produce functional anti-MTB exosomes would dramatically expand the application of exosomes in developing vaccine or drug delivery methods.

## Author Contributions

Y-FS was in charge of research and drafting. JP helped in revision. J-FX was responsible for leading this work and revising the manuscript. All authors contributed to the article and approved the submitted version.

## Funding

This study was supported by the National Natural Science Foundation of China (881870016, 1570009, and 81273237), the Natural Science Foundation of Guangdong Province (2015A030313513 and 2020A1515010283), and the Science and Technology Innovation Fund of Guangdong Medical University (STIF201110, B2012078).

## Conflict of Interest

The authors declare that the research was conducted in the absence of any commercial or financial relationships that could be construed as a potential conflict of interest.
